# Chemoresistance Is Associated with MUC1 and Lewis y Antigen Expression in Ovarian Epithelial Cancers

**DOI:** 10.3390/ijms140611024

**Published:** 2013-05-24

**Authors:** Danye Zhang, Jian Gao, Liancheng Zhu, Zhenhua Hu, Rui Hou, Shuice Liu, Mingzi Tan, Juanjuan Liu, Bei Lin

**Affiliations:** Department of Obstetrics and Gynecology, Shengjing Hospital Affiliated to China Medical University, Shenyang 110004, China; E-Mails: zhangdanye624@163.com (D.Z.); tbagd0519@sina.com (J.G.); zhulc@sj-hospital.org (L.Z.); huzh5062012@163.com (Z.H.); hourui980221@sina.com (R.H.); liusc@sj-hospital.org (S.L.); kitefly1102@126.com (M.T.); juanjuanliu_lg@yahoo.com.cn (J.L.)

**Keywords:** ovarian epithelial cancer, MUC1, Lewis y antigen, chemoresistance, immunohistochemistry

## Abstract

**Objective:**

The aim of this study was to analyze the correlation and clinical significance between the expression of Mucin-1 (MUC1) and the Lewis y antigen with chemoresistance in ovarian epithelial cancers.

**Methods:**

Ovarian cancer patients (*n* = 92) treated at our hospital from May 2005 to July 2009 were divided, according to their treatment and follow-up outcomes, into a resistant group (*n* = 37) or sensitive group (*n* = 55). The expression of MUC1 and Lewis y antigen in ovarian cancer tissues was detected using immunohistochemistry and correlated with chemoresistance.

**Results:**

The positive rates of MUC1 and Lewis y antigen in the resistant group were both 91.89%, significantly higher than their positive rates in the sensitive group (65.45% and 69.09%, respectively, and both *p <* 0.05). MUC1 or Lewis y expression and the pathological stage of the tissue were independent risk factors for chemoresistance (all *p <* 0.05).

**Conclusion:**

The increased expression of MUC1 and the Lewis y antigen is a significant risk factor for chemoresistance in patients with ovarian epithelial cancer.

## 1. Introduction

Ovarian cancer is a critical malignancy with the highest mortality in the female reproductive system. Common treatments for ovarian cancer include surgery and subsequent chemotherapy with drugs based on paclitaxel and cisplatin. Unfortunately, up to 25% of ovarian cancer patients develop chemoresistance, leading to a poor prognosis and a very high mortality rate [1]. Although mechanisms of chemoresistance in ovarian cancers are unclear, cell adhesion mediated-drug resistance (CAM-DR) is believed to play an important role [2,3].

Mucin-1 (MUC1), a type I cross-membrane glycoprotein associated with cell adhesion and migration, is aberrantly expressed in various epithelial cancers [4,5]. We found that MUC1 can be modified by the Lewis y antigen and speculate that fucosylated MUC1 may affect chemoresistance (the results are to be published). Our previous research has also shown that the modification of integrin α5β1 with the Lewis y antigen can increase the adhesion of ovarian cancer cells and its chemoresistance to cisplatin-based drugs [6].

In this study, the expression of MUC1 and the Lewis y antigen in ovarian cancer tissues was detected using immunohistochemistry and, subsequently, correlated with tumor chemoresistance and patient prognosis.

## 2. Results

### 2.1. Expression of MUC1 and the Lewis y Antigen in Ovarian Cancer

**Figure 1:**
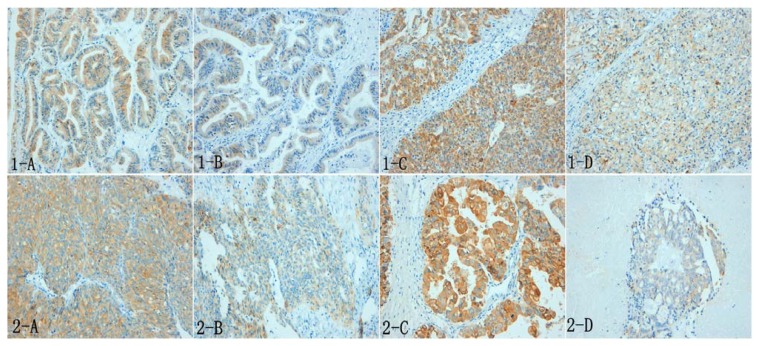
The expression of both MUC1 and the Lewis y antigen in ovarian carcinoma tissues primarily localizes to the cell membrane and cytoplasm [(**1**) MUC1; (**2**) Lewis y antigen; (**A**,**C**) chemotherapy-resistant group; (**B**,**D**) chemotherapy-sensitive group]. All images are magnified 200×.

Among the 92 cases of ovarian cancer, MUC1 expression is primarily located on the cell membrane and cytoplasm. The positive rate of MUC1 in the resistant group (91.89%) is also significantly higher than the sensitive group (69.09%) (*p <* 0.05). Similarly, the Lewis y antigen is primarily localized on the cell membrane and cytoplasm. The positive rate of the Lewis y antigen in the resistant group (91.89%) is also significantly higher than the sensitive group (65.45%) (*p <* 0.05, Table 1, Figure 1).

### 2.2. The Association between MUC1 and Lewis y Antigen Expression

**Figure 2:**
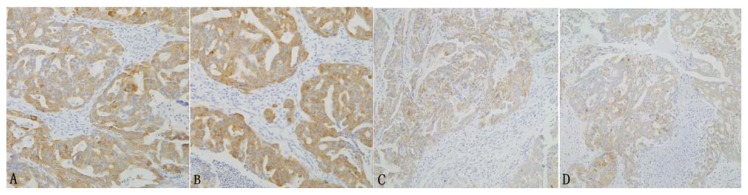
The relationship of the expression of MUC1 and the Lewis y antigen in ovarian carcinoma tissues [(**A**,**C**) the expression of MUC1; (**B**,**D**) the expression of the Lewis y antigen in the same tissue of A and C].

Spearman’s rank correlation analysis shows that MUC1 protein expression positively correlates with Lewis y antigen expression in ovarian epithelial cancer (*r*_s_ = 0.26, *p <* 0.05) (Figure 2).

### 2.3. Risk Factors for Chemoresistance in Ovarian Cancer

Univariate analysis shows that pathological stage, lymph node metastasis and residual tumor size significantly differ between the two groups (all *p <* 0.05), with no significant difference found for pathological type and grade between the two groups (*p >* 0.05) (Table 2). We performed a multivariate logistic regression analysis using the FORWARD stepwise method to consider age, stage, grade, pathological subtype, metastasis of lymph nodes and residual tumor size with MUC1 and the Lewis y antigen serving as covariates and drug resistance or sensitivity as dependent variables. The results show that the stage and expression of MUC1 and the Lewis y antigen were independent risk factors for chemoresistance in ovarian cancer (Table 3).

### 2.4. Prognostic Analysis

**Figure 3:**
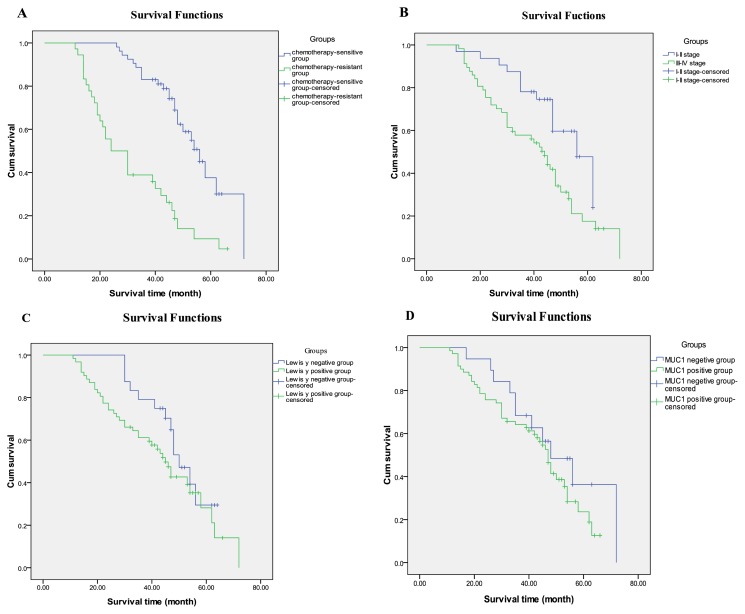
Kaplan-Meier curves (for overall survival) stratified by grouping, using cancer-related deaths as the endpoints. (**A**) Overall survival curves stratified by grouping, *n* = 92, *p* < 0.001; (**B**) overall survival curves stratified by stage, *p* = 0.009; (**C**) overall survival curves stratified by the expression of Lewis y, *p* = 0.139; (**D**) overall survival curves stratified by the expression of MUC1, *p* = 0.256.

Both groups were followed until November 2012. Cox analysis shows that Lewis y antigen expression and pathological stage are independent risk factors for prognosis in ovarian cancer patients (Table 4). Kaplan-Meier analysis shows that the mortality rate is significantly higher in the resistant group (and patients with tumors in pathological stages III–IV) compared with the sensitive group (and patients with tumors in pathological stages I–II) (*p* < 0.001, *p =* 0.009). The mortality rate is higher (although not a statistically significant difference) in patients with both MUC1 and Lewis y antigen expression compared with patients without either MUC1 or Lewis y antigen expression (Figure 3). In addition, the mortality rate did not significantly differ between groups by pathological differentiation or pathological subtype.

## 3. Discussion

MUC1 is aberrantly upregulated in various malignant epithelial cancers, including ovarian cancer [7–9], and is associated with chemoresistance in cancer cells. For example, it has been shown that breast cancer cells expressing MUC1 have a poor response to chemotherapy [10]. The elevation of serum CA153 also suggests a poor responsiveness to initial chemotherapy [11]. Blocking MUC1 can improve the sensitivity of ovarian cancer cells to paclitaxel and doxorubicin [12], as the anti-apoptotic effects of MUC1 on oxidative stress-induced apoptosis [13,14] and genotoxic agent-induced apoptosis [13,15,16] have been correlated to its chemoresistant mechanisms. The absence of apoptosis in cancer cells may lead to chemotherapeutic and radiotherapeutic resistance, resulting in metastasis and a poor prognosis [17].

Ren *et al.* found that the increased expression of MUC1 can induce resistance to genotoxic agents by blocking the intrinsic apoptotic pathway through its C-terminal subunit (MUC1-C) located in the mitochondria [16]. In addition, a reduction in MUC1 proteins can increase chemotherapeutic sensitivity in thyroid cancer [18]. Further studies have proved that MUC1 binds to BAX and blocks the BAX-induced release of cytochrome c from the mitochondria [19]. Furthermore, Raina *et al.* showed that MUC1 binds directly to c-Abl, blocking the localization of c-Abl to the nucleus [15]. MUC1 can also promote p53-dependent cell growth arrest and inhibit p53-dependent apoptosis [20]. Therefore, MUC1 overexpression can attenuate many signals that induce an apoptotic response, leading to poor responsiveness to chemotherapy in cancer cells. Changes in MUC1 structure are also involved in a variety of malignant tumor biology progress. Our previous research has found that upregulation of the Lewis y antigen, a structural component of MUC1, can increase the expression of both MUC1 mRNA transcripts and proteins (the results are to be published). The Lewis y antigen can also increase the tyrosine phosphorylation of MUC1 receptors in ovarian cancer by activating downstream β-catenin, PI3K/Akt and ERK/MAPK signaling pathways associated with apoptosis and chemoresistance [21,22]. Thus, we speculate that modifications in the Lewis y antigen are responsible for its influence on MUC1 in malignant tumors.

MUC1 is a transmembrane protein connected with other transmembrane proteins. MUC1 decreases the adhesion between cancer cells and the adhesion between cancer cells and the extracellular matrix by altering the cytoskeleton [23], thus promoting the release of cancer cells from the primary lesion [23,24]. In addition, MUC1 specifically binds to ICAM-1 on the surface of endothelial cells, increasing the adhesion between heterogeneous cells and leading to invasion of the cancer cells into the vascular wall with subsequent metastasis [23]. Highly metastatic cancer cells are usually very resistant to chemotherapy [22,25], as further shown in our study (more lymph node metastasis in the resistant group, *p* = 0.01). This likely reflects several features of metastatic cancer cells, including their increased invasiveness, decreased apoptosis and increased cell adhesion outside the primary lesion.

In recent years, CAM-DR has been proposed as a novel mechanism of chemoresistance in cancer cells. The higher adhesion between cancer cells, especially metastasized cancer cells, is associated with increased chemoresistance. The glycosylation of proteins is also important, as it can impact protein structure and function. Our previous studies found that an upregulated expression of the Lewis y antigen can increase the expression of CD44, α5β1 and αvβ3. The Lewis y antigen, a structural component of CD44, α5β1 and αvβ3, increases cell adhesion and chemoresistance through various signaling pathways [6,26]. The Lewis y antigen, an important structural component of many molecules, can increase the invasiveness, adhesion and anti-apoptotic nature of cancer cells, leading to an increased chemoresistance.

This is the first study to show that the expression of MUC1 and the Lewis y antigen are increased in chemoresistant ovarian cancer. It also identifies MUC1, Lewis y antigen expression and pathological stage as independent risk factors for chemoresistance in ovarian cancer. The expression of MUC1 positively correlates with the Lewis y antigen (*r*_s_ = 0.26, *p <* 0.05). We have previously shown that the Lewis y antigen is associated with multi-drug resistance in ovarian cancer [27]. Although no statistically significant difference in mortality was found between patients that differ in MUC1 and Lewis y antigen expression, this needs to be clarified with a longer follow-up.

Raluca AB *et al.* have proven that elevated serum MUC1 levels (at baseline or during treatment) were associated with an increased risk of death and serve as an important prognostic factor. MUC1 (CA153) can be used to predict chemoresistance to cisplatin in ovarian cancer [1]. In this study, we increased the sample size, expanded the pathological type, added a group sensitive to chemotherapy and confirmed that MUC1 expression is a reliable index for predicting chemoresistance in ovarian cancer. What’s more, we suppose the underlying mechanism is that, as a glycosylation modified structure of MUC1, Lewis y antigen can affect chemoresistance in multiple ways in which increasing the expression of MUC1 and activating its downstream signals is an important one. Therefore, we believe that the elevated expression of Lewis y showed more significance to predict the chemoresistance of ovarian cancer.

## 4. Materials and Methods

### 4.1. Patients

From May 2005 to July 2009, 92 ovarian cancer patients were treated at our hospital and divided, according to their treatment and follow-up outcomes, into a resistant group (*n* = 37) or sensitive group (*n* = 55). The expression of MUC1 and the Lewis y antigen in ovarian cancer tissues was detected using immunohistochemistry and correlated with the chemoresistance of the patients. All cases consisted of primary ovarian epithelial cancers. The pathological types included serous carcinoma (57 cases), mucinous carcinoma (eight cases), clear cell carcinoma (seven cases), endometrioid carcinoma (six cases) and poorly differentiated adenocarcinoma (12 cases). The patients were pathologically staged according to the FIGO criteria as stage I (19 cases), stage II (15 cases), stage III (56 cases) and stage IV (two cases). The pathological grading rated tumors as well differentiated (14 cases), moderately differentiated (40 cases) and poorly differentiated (38 cases). All patients had primary cancers naïve to radiotherapy and chemotherapy prior to surgery, and their chemotherapy was all taxane combined platinum. The clinical and pathological information of the patients were complete.

The patients were divided, according to NCCN guidelines, into a resistant or sensitive group. The resistant group, initially treated with chemotherapy based on carboplatin and paclitaxel, achieved clinical remission, but had cancer recurrence during the late stage of chemotherapy or within 12 months post-chemotherapy. The sensitive group included patients with clinical remission over 12 months. The clinical features of relapsed ovarian cancer include: (1) persistent and elevated levels of CA125, (2) abdominal masses found by gynecological examination, (3) masses found by clinical imaging studies, (4) ascites and (5) an intestinal obstruction of unknown etiology. The mean age of the patients was 53.94 years, ranging from 24 to 78 years. The median age of the resistant group was 55 years, ranging from 34 to 76 years. The median age of the sensitive group was 52 years, ranging from 24 to 78 years. No significant differences in age were found between the two groups (*p >* 0.05).

### 4.2. Immunohistochemistry Staining and Scoring

Ovarian cancer tissue samples were cut into 5 μm sections, and three sections used for each sample. The expression of MUC1 and the Lewis y antigen were detected by the streptavidin-peroxidase method. Breast cancer tissues served as positive controls, while sections treated with phosphate buffered saline instead of primary antibodies were used as negative controls. The primary antibodies used were mouse anti-human antibodies against MUC1 (1:320 dilution, purchased from Santa Cruz, CA, USA) and the Lewis y antigen (1:160 dilution, purchased from Abcam Co, Cambridge, UK). Staining was performed according to the manufacturer’s instructions.

The staining results were scored according to the immunoreactive intensity of the cell membrane and cytoplasm (no staining = 0, light yellow = 1, brown yellow = 2 and dark brown = 3. Positive cells were also counted under 400× magnification and scored (positive rate <5% = 0, 5%–25% = 1, 26%–50% = 2, 51%–75% = 3, >75% = 4). The scores for immunoreactive intensity and positive rate were multiplied and the results used to rate the pathological examination (0–2 as negative (−), 3–4 as weakly positive (+), 5–8 as mildly positive (++) and 9–12 as strongly positive. Two pathologists independently read the sections and made the scoring decisions.

### 4.3. Statistical Analysis

The data were statistically analyzed using SPSS 17.0. Categorical data were analyzed with the Chi-square (χ^2^) test and continuous data with the *t*-test. Chemoresistance-related factors were analyzed with multivariate logistic regression and their relationships analyzed with Spearman’s rank correlation coefficient. Relationships with prognosis were analyzed with the Cox model. *p <* 0.05 was considered statistically significant.

## 5. Conclusions

An increase in MUC1 expression is associated with the chemoresistance of ovarian cancer; thus the expression of MUC1 can be used to predict ovarian cancer chemoresistance. The Lewis y antigen increases MUC1 expression, thereby increasing the invasive and anti-apoptotic nature of cancer cells and their resistance to cytotoxic chemotherapies. The Lewis y antigen also alters the expression and conformation of adhesion molecules, inducing chemoresistance in cancer cells by activating these associated signaling pathways.

## Figures and Tables

**Table 1:** The expression of Mucin-1 (MUC1) and the Lewis y antigen in chemoresistant and chemosensitive groups.

Group	*N*	MUC1					Lewis y				
	
		−	+	++	+++	Positive (%)	−	+	++	+++	Positive (%)
	
Resistant	37	3	7	13	13	34/37 (91.89)	3	13	18	8	34/37 (91.89)
Sensitive	55	17	3	16	19	38/55 (69.09)	19	13	16	0	36/55 (65.45)

**Table 2:** Univariate analysis of factors associated with chemoresistance.

Group	*N*	Resistant		Sensitive		*p*
	
		*n* = 37	%	*n* = 55	%	
Stage						
I	19	1	2.7	18	32.7	0.001
II	15	3	8.1	12	21.8	
III	56	32	86.5	24	43.6	
IV	2	1	2.7	1	1.8	

Tumor grade						
I	14	4	10.8	9	14	0.091
II	40	14	37.8	25	40	
III	38	19	51.4	21	38	

Pathological subtype						
Serous carcinoma	57	24	64.9	33	60	0.339
Mucinous carcinoma	8	5	13.5	3	5.5	
Endometrioid adenocarcinoma	6	1	2.7	5	9.1	
Clear cell carcinoma	7	1	2.7	6	10.9	
Poorly differentiated adenocarcinoma	12	5	13.5	7	12.7	
Undifferentiated carcinoma	2	1	2.7	1	1.8	

Lymph node metastasis						
Yes	11	3	8.1	8	14.5	0.001
No	50	11	29.7	39	70.9	
Unknown	31	18	48.6	13	23.6	

Residual tumor size						
≤1 cm	42	8	21.6	36	65.5	0.001
1–2 cm	13	7	18.9	6	10.9	
≥2 cm	13	10	27.0	3	5.5	

**Table 3:** Multivariate analysis of factors associated with chemoresistance.

Type	β-value	Sx	*p*	OR * value	95% CI
MUC1	1.905	0.905	0.035	6.716	1.139 39.604
Lewis y	1.245	0.341	0.000	3.474	1.781 6.776
Stage	2.048	1.280	0.003	7.753	1.990 30.203

* OR = Odds ratio.

**Table 4:** Cox analysis of prognosis in ovarian cancer patients.

T	β-value	SE	*p*	Exp (β)	95%CI
Stage	0.774	0.335	0.021	2.168	1.125 4.176
Lewis y	0.320	0.143	0.025	1.377	1.040 1.823
